# PB.22: Does the Nottingham predictor of bidimensional product of calcium accurately predict the success of wide local excision for ductal carcinoma *in situ *surgery in our centre?

**DOI:** 10.1186/bcr3522

**Published:** 2013-11-08

**Authors:** R Gray, G Porter, J Steel

**Affiliations:** 1 Plymouth Hospitals NHS Trust, Plymouth, UK

## Introduction

A 2007 Nottingham paper suggests that a mammographic bidimensional product (BDP) of <800 mm^2 ^calcification in high-grade ductal carcinoma *in situ *(DCIS) and <400 mm^2 ^in low/intermediate DCIS is a powerful predictor of successful excision. We wanted to see whether their results were reproducible at our centre.

## Methods

Eighty-four consecutive cases of DCIS over 18 months were reviewed and examined to determine the BDP of calcium (extent of mammographic calcification was measured in two orthogonal planes on the oblique view). Was reoperation required? Does the pathology report show clear margins? We excluded 15 patients that had a mastectomy (18%).

## Results

See Table 1.

**Table 1:** 

BDP	Successful WLE	Margins not clear
High-grade DCIS		
<800 mm^2^	15, 60% (Nottingham 69%)	10
>800 mm^2^	2, 50% (Nottingham 24%)	2
Low-grade DCIS		
<400 mm^2^	24, 86% (Nottingham 73%)	4
>400 mm^2^	9, 75% (Nottingham 33%)	3

## Conclusion

We did not obtain similar results to the Nottingham paper. The success rate of WLE for low-grade DCIS was not (significantly) affected by BDP. Had BDP led to a recommendation not to perform WLE, the majority (75%) of women in this low-risk group, most at risk of overtreatment, would have had a mastectomy when conservative surgery would have succeeded. While we have not the large numbers of the Nottingham paper, we feel that in our centre at least, BDP of calcification seems not to be a practically useful predictor of excision success in nonhigh-grade DCIS.

